# A Nuclear Localization of the Infectious Haematopoietic Necrosis Virus NV Protein Is Necessary for Optimal Viral Growth

**DOI:** 10.1371/journal.pone.0022362

**Published:** 2011-07-21

**Authors:** Myeong Kyu Choi, Chang Hoon Moon, Myoung Seok Ko, Unn-Hwa Lee, Wha Ja Cho, Seung Ju Cha, Jeong Wan Do, Gang Joon Heo, Soo Geun Jeong, Yoo Sik Hahm, Abdallah Harmache, Michel Bremont, Gael Kurath, Jeong Woo Park

**Affiliations:** 1 Department of Biological Sciences, University of Ulsan, Ulsan, Korea; 2 Biomedical Research Center, Ulsan University Hospital, College of Medicine, University of Ulsan, Ulsan, Korea; 3 South and West Sea Fisheries Research Institute, National Fisheries Research and Development Institute, Yeosu, Korea; 4 College of Veterinary Medicine, Chungbuk National University, Cheongju, Korea; 5 Ulsan Institute of Health and Environment, Ulsan, Korea; 6 Unite de Virologie and Immunologie Moleculaires, INRA CRJ, Domaine de Vilvert, Jouy en Josas, France; 7 US Geological Survey, Western Fisheries Research Center, Seattle, Washington, United States of America; Nanyang Technological University, Singapore

## Abstract

The nonvirion (NV) protein of infectious hematopoietic necrosis virus (IHNV) has been previously reported to be essential for efficient growth and pathogenicity of IHNV. However, little is known about the mechanism by which the NV supports the viral growth. In this study, cellular localization of NV and its role in IHNV growth in host cells was investigated. Through transient transfection in RTG-2 cells of NV fused to green fluorescent protein (GFP), a nuclear localization of NV was demonstrated. Deletion analyses showed that the ^32^EGDL^35^ residues were essential for nuclear localization of NV protein, and fusion of these 4 amino acids to GFP directed its transport to the nucleus. We generated a recombinant IHNV, rIHNV-NV-ΔEGDL in which the ^32^EGDL^35^ was deleted from the NV. rIHNVs with wild-type NV (rIHNV-NV) or with the NV gene replaced with GFP (rIHNV-ΔNV-GFP) were used as controls. RTG-2 cells infected with rIHNV-ΔNV-GFP and rIHNV-NV-ΔEGDL yielded 12- and 5-fold less infectious virion, respectively, than wild type rIHNV-infected cells at 48 h post-infection (p.i.). While treatment with poly I∶C at 24 h p.i. did not inhibit replication of wild-type rIHNVs, replication rates of rIHNV-ΔNV-GFP and rIHNV-NV-ΔEGDL were inhibited by poly I∶C. In addition, both rIHNV-ΔNV and rIHNV-NV-ΔEGDL induced higher levels of expressions of both IFN1 and Mx1 than wild-type rIHNV. These data suggest that the IHNV NV may support the growth of IHNV through inhibition of the INF system and the amino acid residues of ^32^EGDL^35^ responsible for nuclear localization are important for the inhibitory activity of NV.

## Introduction

Infectious hematopoietic necrosis virus (IHNV) is a rhabdovirus belonging to the genus *Novirhabdovirus* of the *Rhabdoviridae* family. IHNV causes an acute disease in wild and hatchery-reared salmonid fish in North America, Europe, and Asia [1], [2]. Similar to other rhabdoviruses, the IHNV genome encodes five structural proteins that make up the virions: a nucleoproetin (N), a polymerase-associated phosphoprotein (P), a matrix protein (M), a unique glycoprotein (G), and a RNA polymerase protein (L) [3], [4]. The Novirhabdovirus genome possesses an additional non-virion (NV) gene located between the viral glycoprotein (G) and the polymerase (L) genes [3]. While the functions of most IHNV proteins are known, that of the NV protein remains unclear. Recently, Biacchesi et al [5] demonstrated that a recombinant IHNV (rIHNV) knockout with no NV gene was severely impaired for growth in cell culture and had reduced pathogenicity in rainbow trout [6], suggesting a crucial role of NV for optimal IHNV replication. However, the molecular basis for the role of NV is not known.

Virus-infected cells synthesize and secrete type I interferons (IFNs) which are considered the first line of host defense against viral infection [7], [8]. Secreted IFNs stimulate susceptible cells to express more than 300 IFN-stimulated genes (ISGs), whose concerted action leads to limiting further viral growth and spread [8]. The myxovirus resistance gene Mx, the PKR protein kinase stimulated by dsRNA, and the 2′-5′ oligoadenylate synthetase (OAS) are among the best-characterized antiviral ISGs [9]. The IFN system has also been found in teleost fish including Atlantic salmon and rainbow trout. Teleost fish IFNs can induce the expressions of several ISGs with antiviral activity [10], [11]. The IFN system induced by poly I∶C treatment [12], [13] limited the growth of IHNV in cell culture [14] and recombinant IFN treatment protected rainbow trout from IHNV infection [15], suggesting that IHNV replication is highly sensitive to the IFN-induced antiviral responses of host cells.

It is now evident that most viruses have evolved means to down-regulate IFN responses. In many cases they use nonstructural viral proteins for that purpose. A good example is the nonstructural protein NS1 of the influenza A virus. NS1 binds to RIG-I/IPS-1 complexes [16], [17], [18] and blocks downstream signaling [19], [20], [21], resulting in attenuation of type I IFN and inflammatory cytokine expression. These anti-interferon activities of NS1 are possible due to a complex regulation of NS1 cellular localization mediated by two nuclear localization signals (NLSs) and one nuclear export signal (NES), which allow the transport of this protein between the cytoplasm and nucleus of the infected cell [22], [23]. Rhabdoviruses also have the ability to limit IFN production and IFN signaling [24]. However, they have been reported to use structural proteins for this purpose. In mammalian rhabdoviruses, the M protein of vesicular stomatitis virus (VSV) and the P protein of rabies virus (RV) have been shown to inhibit host IFN-mediated responses using different molecular mechanisms [25], [26], [27], [28], [29], [30], [31], [32], [33], [34], [35]. However, until now, it is not known how IHNV interacts with the host IFN system.

In this study, we showed that the 111-amino acid (aa) NV protein of IHNV can localize to the nucleus, and identified amino acid residues, ^32^EGDL^35^, within the NV protein that are responsible for the nuclear localization of the NV protein. We then investigated the roles of the NV protein and the ^32^EGDL^35^ residues in IHNV growth using a series of various recombinant IHNV. Collectively, our data suggest that the NV can support the growth of IHNV in host cells through inhibition of the induction of IFN systems, and that the amino acid sequence ^32^EGDL^35^, responsible for nuclear localization of the NV, plays important role in the inhibitory activity of NV.

## Methods

### Cells and viruses

CHSE-214 (chinook salmon embryo) (ATCC CRL-1681), RTG-2 (rainbow trout gonad) (ATCC CCL-55), and EPC (epithelioma papulosum cyprini) (ATCC CRL-2872) cells were grown at 18°C in Eagle's minimal essential medium (MEM) supplemented with 10% fetal bovine serum (FBS). The transgenic cell line, RTG-P1 [36], was purchased from ATCC (CRL-2829) and was cultivated in Eagle's MEM supplemented with 10% FBS and 200 µg/ml Neomycin (G418, Sigma, St. Louis, MO). The IHNV-PRT strain used in this study is a 1991 isolate from rainbow trout Pyongchang Korea [37] at passage level 35. The virus was propagated in EPC cells at 16°C and quantified in terms of plaque forming units (PFU/ml) using the standard plaque assay [38].

### Effect of poly I∶C on IHNV growth

To determine the effect of pretreatment of cells with poly I∶C on the growth of IHNV, 25 cm^2^ culture flasks of RTG-2 cells were inoculated with poly I∶C (final concentration 25 µg/ml) (P1530, Sigma) and incubated for 24 h. The medium was removed and replaced with fresh medium without poly I∶C, and the cells were challenged with IHNV at an MOI of 0.01 PFU/cell. Samples of the supernatant were collected at 0, 24, and 48 h after virus infection and were stored at −80°C until use.

To assess the effect of poly I∶C treatment after viral infection, RTG-2 cells were challenged with IHNV at an MOI of 0.01 and were incubated for 24 h at 16°C. The medium was removed and replaced with fresh medium with or without poly I∶C (25 µg/ml). Supernatant samples were collected at 0, 24, and 48 h after poly I∶C treatment and were stored at −80°C until use.

### Plasmids expressing NV and transfections

The full-length cDNA of NV was amplified using RT-PCR from the RNA of CHSE-214 cells infected with IHNV-PRT using PCR primers as follows: pcDNA-NV: 5′-GGATCCATGGACCACCGCGAAACAAAC-3′, 5′-CTCGAGTCTGGGATAAGCAAGAAA-3′; pEGFP-NV: 5′-GCTAGCATGGACCACCGCGAAACAAAC-3′, 5′-GGATCCAATCTGGGATAAGCAAGAAA-3′. The PCR products were subcloned into the *BamH*I/*Xho*I site of pcDNA6/V5 (Invitrogen, Carsbad, USA) or *Nhe*I/*BamH*I site of pEGFP-N1 (Clontech, Mountain View, CA) to create pcDNA6/V5-NV_PRT_ and pEGFP/NV(1–111), respectively.

A variety of deletion mutants of NV were PCR amplified using pEGFP/NV(1–111) as a template with the following primer pairs: NV(1–37): 5′-GCTAGCATGGACCACCGCGAAACAAAC-3′, 5′-GGATCCAAGCCCCATACCAGGTCTCC-3′; NV(38–73): 5′-GCTAGCATGTGTGAAGAGGACGACGCA-3′, 5′-GGATCCAACTTGGTGATGCTGAGGTC-3′; NV(74–111): 5′-GCTAGCATGGAGGGGCATCTACTTTTT-3′, 5′-GGATCCAATCTGGGATAAGCAAGAAA-3′; NV(1–20): 5′-GCTAGCATGGACCACCGCGAAACAAAC-3′, 5′-GGATCCTTGTATCGCAGAACTTC-3′; NV(21–35): 5′- CTAGCATGAACGAGGTGGCCGGACACGGCTTCCTCTTTAAGGAGGGAGACCTGCCG-3′, 5′-GATCCGGCAGGTCTCCCTCCTTAAAGAGGAAGCCGTGTCCGGCCACCTCGTTCATG-3′; NV(ΔEGDL): 5′-GCTAGCATGGACCACCGCGAAACAAAC-3′, 5′-CACACCATACCTTAAAGAGGAAGCCGTGTC-3′, 5′-CCTCTTTAAGGTATGGTGTGAAGAGGACGA-3′, 5′-GGATCCAATCTGGGATAAGCAAGAAA-3′. PCR products were inserted into the *Nhe*I/*BamH*I site of pEGFP-N1.

To generate the plasmid constructs pEGFP/NV(31–35) and pEGFP/NV(^32^EGDL^35^) and to mutate the ^32^EGDL^35^ into ^32^AGDL^35^, ^32^EADL^35^, ^32^EGAL^35^, or ^32^EGDA^35^, the following oligonucleotides were synthesized at Integrated DNA Technologies (Coralville, IA), annealed, and ligated into the *Nhe*I/*BamH*I site of pEGFP-N1 (CLONTECH, Inc.): NV(31–35): 5′-CTAGCATGAAGGAGGGAGACCTGCCG-3′, 5′-GATCCGGCAGGTCTCCCTCCTTCATG-3′; ^32^EGDL^35^: 5′-CTAGCATGGAGGGAGACCTGCCG-3′, 5′-GATCCGGCAGGTCTCCCTCCATG-3′; ^32^AGDL^35^: 5′-CTAGCATGGCGGGAGACCTGCCG-3′, 5′-GATCCGGCAGGTCTCCCGCCATG-3′; ^32^EADL^35^: 5′-CTAGCATGGAGGCAGACCTGCCG-3′, 5′-GATCCGGCAGGTCTGCCTCCATG-3′; ^32^EGAL^35^: 5′-CTAGCATGGAGGGAGCCCTGCCG-3′, 5′-GATCCGGCAGGGCTCCCTCCATG-3′; ^32^EGDA^35^: 5′-CTAGCATGGAGGGAGACGCGCCG-3′, 5′-GATCCGGCGCGTCTCCCTCCATG-3′.

To express the proteins *in vitro*, CHSE-214 and RTG-2 cells were electroporated with various types of pEGFP/NV constructs using the Neon™ Transfection System (Invitrogen, Carlsbad, CA) according to the manufacturer's instructions.

### Subcellular fractionation

Forty-eight hours after transfection with the pEGFP/NV(1–111) constructs, CHSE-214 cells were harvested, and nuclear and cytosolic fractions were prepared from 5×10^7^ CHSE-214 cells using the Subcellular Proteome Extraction kit (ProteoExtract™, Calbiochem, Darmstadt, Germany) according to the manufacturer's instructions.

### SDS-PAGE and Western blots

Cells were washed twice with cold PBS, and 30–50 µg of protein was resolved using SDS-PAGE, transferred onto Hybond-P membranes (GE Healthcare, Piscataway, NJ), and probed with appropriate dilutions of the anti-GFP (sc-9996, Santa Cruz Biotechnology, Santa Cruz, CA) or anti-V5 antibody (Genentech, San Francisco). Immunoreactivity was detected using an ECL detection system (GE Healthcare). Autoradiography films were exposed at multiple time points to ensure that the images were not saturated.

### Confocal microscopy

After transfection with 10 µg of various types of pEGFP/NV constructs, cells were seeded on confocal dishes (SPL-200350, SPL Life Sciences, Pocheon, Korea). On the following day, cells were observed with a FluoView™ 500 confocal microscope (Olympus, Tokyo, Japan).

### Viral growth analysis

Infections were carried out in 25 cm^2^ culture flasks containing confluent monolayers of RTG-2 cells at a MOI of 0.01 unless otherwise stated. At the end of 1 h adsorption period at 16°C, the cells were washed three times with MEM containing no serum. Five milliliters of MEM containing 5% FBS was then added to each culture flask and the flasks were incubated at 16°C. Samples of 200 µl of medium were collected at 0, 24, and 48 h post-infection (p.i.), and stored at −80°C. Viral titers were determined for all samples, in duplicate, using a plaque assay on EPC cells as described above.

### Mx promoter-reporter assay

To confirm the presence and to quantify IFN in culture supernatants, we used the transgenic RTG-P1 cell line that contains a stable insertion of the IFN-responsive Mx promoter linked to a luciferase reporter gene [36]. Culture supernatant samples were collected from RTG-2 cells at 24 h p.i. and then incubated at 37°C for 2 h to inactivate the virus. One milliliter of culture supernatant sample was added to each of three wells in a 24-well plate containing the transgenic RTG-P1 cells, and incubated for 48 h. As a positive control, RTG-P1 cells were treated with poly I∶C (25 µg/ml) for 24 h. Cells from the wells were rinsed with EMEM-0 and trypsinized. The detached cells were collected using centrifugation, and the pellet was stored at −80°C. To assay the luciferase activity cells were lysed with lysis buffer (Promega, Madison, WI) and mixed with luciferase assay reagent (Promega) and the chemiluminescent signal was measured in a Wallac Victor 1420 Multilabel Counter (EG&G Wallac, Turku, Finland).

### Plasmid construction and recovery of recombinant virus

A full-length antigenomic IHNV cDNA construct plasmid (pIHNV) derived from a French IHNV strain (IHNV 32/87) [5] was used to generate recombinant IHNVs (rIHNV). Coding regions (ORFs) of wild-type NV and NV-ΔEGDL, in which the nucleotides encoding^32^EGDL^35^ were deleted from the NV of the IHNV-PRT strain, were amplified using RT-PCR from pEGFP/NV(1–111) and pEGFP/NV(ΔEGDL), respectively. RT-PCR was performed using the following primer pair: CACGTAAAGTACCAGGTCATC, ACACACCCACAGTATCACT; Unique *Spe*I and *Sma*I restriction enzyme sites surrounding the NV ORFs of pIHNV were created by site-directed mutagenesis with the Quick Change kit (Agilent Technologies, Inc., Santa Clara, CA). The entire NV_32/87_ ORF of pIHNV was deleted using *Spe*I-*Sma*I digestion and replaced with amplified DNA fragments containing the NV_PRT_ or NV_PRT_-ΔEGDL ORFs to generate pIHNV/NV_PRT_ or pIHNV/NV_PRT_(ΔEGDL), respectively. The presence and positions of the inserted genes were confirmed by RT-PCR and restriction enzyme analysis as well as sequencing of the inserted sites in the plasmids. Recombinant viruses were recovered from EPC cells as previously described [5]. To summarize briefly, EPC cells were infected with a vaccinia virus recombinant that expresses T7 RNA polymerase (vTF7-3) [39] and was transfected with the full-length cDNA clones and three support plasmids that expressed the N, P, and L proteins required for RNA encapsidation and replication. Infectious viruses were recovered from the supernatant medium, and were amplified by passage on EPC cells at a low multiplication of infection. Viral titers of recombinant virus stocks were quantified using the plaque assay. We also used two additional rIHNVs as controls: a parental rIHNV generated from a French IHNV strain (IHNV 32/87) and a rIHNV-ΔNV-GFP in which the entire NV ORF was deleted and replaced with a green fluorescent protein (GFP) ORF [5].

### Quantitative real-time and semi-quantitative RT-PCR for Mx1, IFN1 and IHNV G expressions in RTG-2 cells

Total RNA was isolated from infected cells using the Trizol (Invitrogen) extraction method and DNase I-treated total RNA was reverse transcribed using oligo-dT and Superscript II reverse transcriptase (Invitrogen) according to the manufacturer's instructions. Quantitative real-time PCR was performed using SYBR® Green PCR Master Mix (Applied Biosystems, Foster City, CA) and the ABI Prism 7900 HT (Applied Biosystems). Specificities of each primer pair were confirmed via melting curve analysis and agarose-gel electrophoresis. The housekeeping gene, acidic ribosomal phosphoprotein P0 (ARP) was used as a normalizing gene [40], and PCR primer pairs were as follows: Mx1: 5′-GGTTGTGCCATGCAACGTT-3′, 5′-GGCTTGGTCAGGATGCCTAAT-3′; IFN1: 5′-GCGAAACAAACTGCTATTTACAATGTATA-3′, 5′-TCACAGCAATGACACACGCTC-3′; IHNV-G: 5′-GCGCACGCCGAGATAATATCAA-3′, 5′-TCCCGTGATAGATGGAGCCTTT-3′; ARP: 5′-GAAAATCATCCAATTGCTGGATG-3′, 5′-CTTCCCACGCAAGGACAGA-3′.

### Statistical analysis

Differences in the expression of IFN1 and Mx1, as well as Mx1 promoter activity and the viral growth among rIHNVs-infected cells, were evaluated by an unpaired Student's *t*-test (two-tailed). A *P* value<0.05 was considered to indicate statistical significance.

## Results

### Nuclear localization of NV-GFP

**Figure 1:**
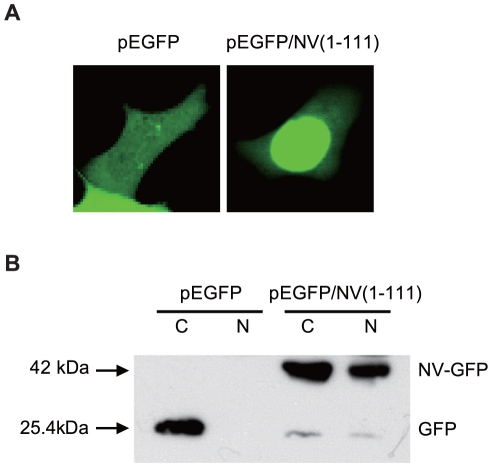
Nuclear localization of NV-GFP proteins in CHSE-214 cells. CHSE-214 cells were transiently transfected with plasmids pEGFP-N1 or pEGFP/NV(1–111) and incubated for 48 h. *A*, The fluorescent signals were examined using confocal microscopy. Each image is representative of the majority of the cells observed in several fields. *B*, Cells were harvested and nuclear and cytoplasmic fractions were prepared as described in the Materials and Methods section. The resulting fractions were analyzed using 10% SDS-PAGE, followed by Western blotting with anti-GFP antibody. C denotes the cytoplasmic fraction, N denotes the nuclear fraction.

The NV of IHNV has been reported to be essential for efficient growth and pathogenicity of IHNV [6]. In an effort to gain further insight into the functions of the NV in viral replication, we first examined the intracellular distribution of the NV. To date there is no commercially available anti-IHNV NV antibody. Therefore, we tagged the NV with GFP by cloning the full-length cDNA of the NV gene into a pEGFP-N1 expression vector, transfected the resulting plasmid [pEGFP/NV(1–111)] into CHSE-214 cells and investigated the subcellular localization of the NV-GFP fusion protein using confocal microscopy. Representative images of cells transfected with plasmids encoding NV-GFP and GFP alone are shown in Figure 1A. As expected, diffuse fluorescence was observed throughout both the cytoplasm and the nucleus in the pEGFP-N1-transfected cells (Figure 1A). However, fusion of the NV protein to GFP altered the distribution of GFP, resulting in the accumulation of fluorescent label in the nucleus and decreased accumulation in the cytoplasm (Figure 1A).

To confirm the confocal microscopy observations, nuclear and cytoplasmic fractions of CHSE-214 cells transfected with the pEGFP-N1 and pEGFP/NV(1–111) plasmids were analyzed using Western blot analysis with anti-GFP antibody (Figure 1B). The presence of NV-GFP fusion protein in both the nuclear and cytoplasmic extracts was generally in agreement with the subcellular distribution observed via fluorescence microscopy. The GFP encoded by the control plasmid without fusion to NV was not detected in the nuclei with Western blotting (Figure 1B). Although the fluorescence signal of the GFP alone appeared evenly distributed throughout the cell, we suspect that the diffuse distribution in the cytoplasm made it appear that this protein was distributed in the nucleus under fluorescence microscopy. These results suggest that the nuclear localization of NV is an intrinsic property of this protein and is independent of other viral factors.

### The amino terminus of NV is required for nuclear localization

**Figure 2:**
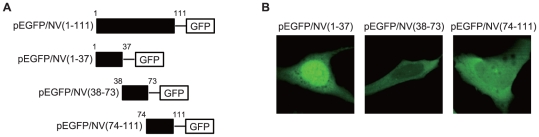
Mapping of the NV region that supports nuclear localization. *A*, Schematic diagram of the full-length NV-GFP fusion protein and truncated mutants containing the N-terminal (NV(1–37)), middle region (NV(38–73)), and C-terminal (NV(74–111)) of the NV protein. *B*, CHSE-214 cells were transiently transfected with mutant NV constructs and examined live 48 h after transfection using confocal microscopy.

The sequence of the NV gene does not encode any canonical nuclear-localization signal (NLS) [41], [42]. Thus to map the protein domains responsible for nuclear localization, three NV fragments, corresponding to amino acid residues (aa) 1 to 37 (NV(1–37)), aa 38 to 73 (NV(38–73)), and aa 74 to 111 (NV(74–111)) of NV, were tagged with GFP (Figure 2A) and were expressed in CHSE-214 cells. Figure 2B shows typical images of cells that are representative of each truncated NV-GFP fusion protein at 48 h after transfection. While both the NV(38–73) and the NV(74–111) were evenly localized in the cytoplasm and the nucleus, the N-terminal fragment NV(1–37) had a nuclear-localization pattern similar to that of full-length NV(1–111) (Figure 2B), indicating that the NV(1–37) alone can mediate nuclear targeting.

### Amino acids ^32^EGDL^35^ are responsible for nuclear localization of NV protein

**Figure 3:**
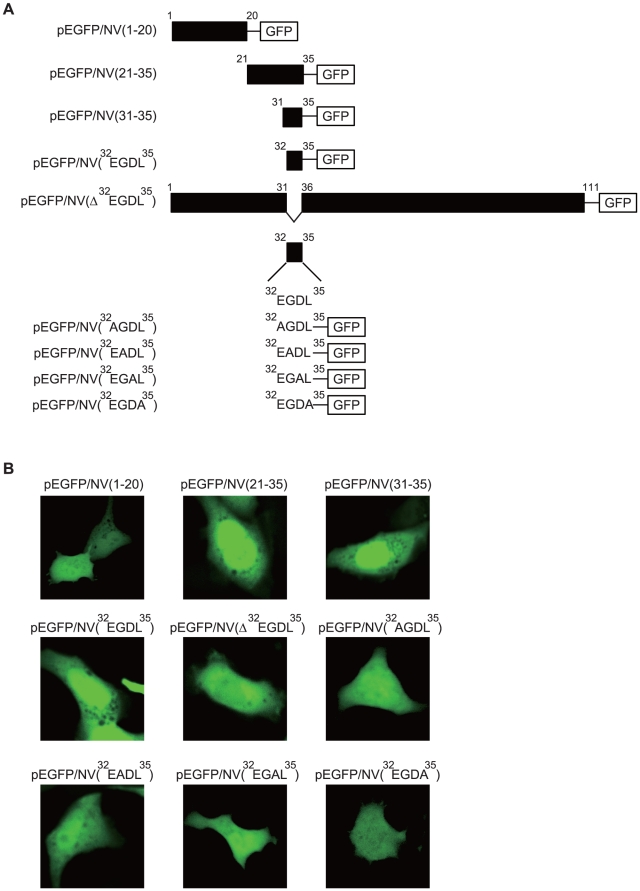
Identification of an amino acid motif responsible for nuclear localization of NV. *A*, Schematic diagram of the GFP fusion proteins used to identify the motif responsible for nuclear localization of NV. The names of the constructs are shown on the left. *B*, CHSE-214 cells were transiently transfected with various types of plasmid constructs and, after incubation for 48 h, fluorescent signals were examined using confocal microscopy. Each image is representative of the majority of the cells observed in several fields.

To more precisely define and characterize the regions within the NV(1–37) responsible for nuclear localization, a series of truncations from either the C or N terminus of NV(1–37) were performed (Figure 3A). Each truncated NV fragment was tagged with GFP and expressed in CHSE-214 cells. Removal of 31 additional residues from the N terminus or two residues from the C terminus of NV(1–37) did not alter its nuclear localization pattern (Figure 3B). Collectively, these results suggest that ^32^EGDL^35^ of NV are sufficient for nuclear translocation since the ^32^EGDL^35^-GFP fusion protein accumulated in the nuclei (Figure 3B).

To determine whether ^32^EGDL^35^ were essential for nuclear localization of NV, they were deleted from the NV-GFP fusion protein (Figure 3A). CHSE-214 cells were transfected with this construct, and the distribution of the NV(Δ^32^EGDL^35^)-GFP mutant proteins was examined 48 h after transfection. As shown in Figure 3B, NV(Δ^32^EGDL^35^)-GFP was not targeted to the nucleus, indicating that amino acid residues ^32^EGDL^35^ are critical for nuclear localization of NV.

To determine which amino acids within ^32^EGDL^35^ are necessary for nuclear localization, each amino acid within ^32^EGDL^35^ was replaced with uncharged alanine and the resultant mutants were expressed as fusion proteins with GFP (Figure 3A). As shown in Figure 3B, all substitutions of amino acid residues within the ^32^EGDL^35^ decreased the accumulation of fluorescent label in the nucleus (Figure 3B). These results suggest that all 4 residues within ^32^EGDL^35^ may be critical for nuclear localization.

**Figure 4:**
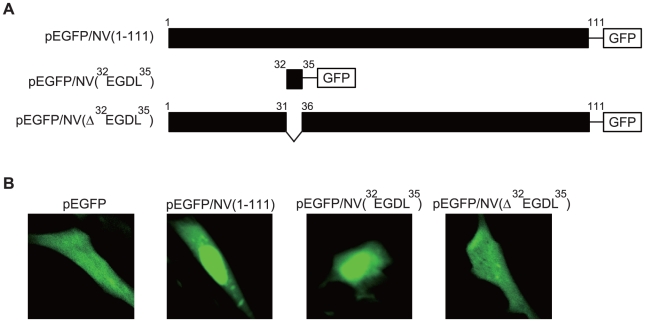
Nuclear localization of NV-GFP protein in RTG-2 cells. *A*, Schematic diagram of the GFP fusion proteins used to transfect RTG-2 cells. *B*, RTG-2 cells were transiently transfected with pEGFP-N1, pEGFP/NV(1–111), pEGFP/NV(^32^EGDL^35^), or pEGFP/NV(ΔEGDL). After incubation for 48 h, fluorescent signals were examined using confocal microscopy. Each image is representative of the majority of the cells observed in several fields.

The nuclear localization of NV was also confirmed in RTG-2 cells using confocal microscopy. While fluorescent signals of the NV-GFP and the ^32^EGDL^35^-GFP were accumulated within the nucleus, those of the NV(Δ^32^EGDL^35^)-GFP were diffused throughout the nucleus and cytoplasm just like those of the GFP (Figure 4). These results suggest that nuclear localization of IHNV NV mediated by the amino acids ^32^EGDL^35^ is common to both CHSE-214 and RTG-2 cells.

### Amino acids ^32^EGDL^35^ are required for the efficient growth of IHNV in RTG-2 cells

**Figure 5:**
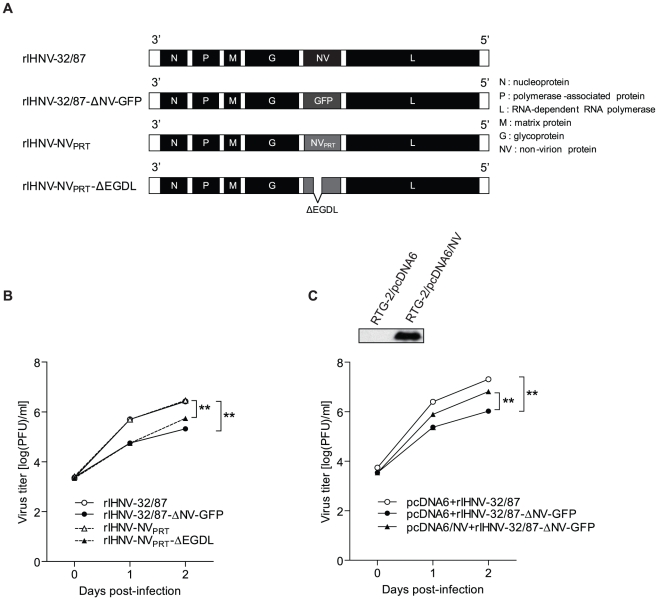
Growths of rIHNV-32/87, rIHNV-32/87-ΔNV-GFP, rIHNV-NV_PRT_, and rIHNV-NV_PRT_-ΔEGDL in RTG-2 cells. *A*, Schematic representation of the recombinant IHNVs used in this study. rIHNV-NV_PRT_, and rIHNV-NV_PRT_-ΔEGDL were generated as described in the Materials and Methods section, and rIHNV-32/87 and rIHNV-32/87-ΔNV-GFP were used as controls. NV_PRT_ refers to the NV gene from the IHNV PRT strain. All other genes are from IHNV 32/87 strain. *B*, Comparative growths of rIHNV-32/87, rIHNV-32/87-ΔNV-GFP, rIHNV-NV_PRT_, or rIHNV-NV_PRT_-ΔEGDL in RTG-2 cells. The cells were infected with rIHNVs at an MOI of 0.01 PFU/cell, and samples of the supernatant medium were collected at 0, 24, and 48 h p.i. The samples were titrated in duplicate using a plaque assay on EPC cells. The results are presented as the means ± SD of three independent experiments (**P<0.01). C. Effect of NV expression on the growth of rIHNV-32/87-ΔNV-GFP in RTG-2 cells. RTG-2 cells were transfected with pcDNA6/V5-NV_PRT_ or empty vector pcDNA6/V5. After incubation for 24 h, the level of NV protein was determined by Western blotting with anti-V5 antibody (top panel). RTG-2 cells transfected with pcDNA6/V5-NV_PRT_ or empty vector were infected with rIHNVs at an MOI of 0.01 PFU/cell and samples of the supernatant medium were collected at 0, 24, and 48 h p.i. The samples were titrated in duplicate using a plaque assay on EPC cells. The results are presented as the means ± SD of three independent experiments (**P<0.01).

To determine whether the amino acids ^32^EGDL^35^ are involved in the growth of IHNV in cell culture, two rIHNVs based on the genomic background of rIHNV-32/87 were generated by replacing the NV of rIHNV-32/87 strain with the wild-type NV of the PRT strain (rIHNV-NV_PRT_) or mutant NV of PRT lacking amino acids ^32^EGDL^35^ (rIHNV-NV_PRT_-ΔEGDL) (Figure 5A). A parental rIHNV-32/87 and a NV-knockout mutant rIHNV-32/87-ΔNV-GFP [5] were used as controls.

The IHNV-PRT and IHNV-32/87 strains were originally isolated from rainbow trout [5], [37] and, thus, we examined growth of the rIHNVs in rainbow trout-derived RTG-2 cells. We infected RTG-2 cells with rIHNVs at an MOI of 0.01 and infectious virus titers shed into the media were determined via plaque assay. Figure 5B illustrates a comparison of the rIHNV yields at 0, 24, and 48 h post-infection (p.i.) in RTG-2 cells, presented as averages from three independent experiments. Replacement of the NV gene of rIHNV-32/87 with the NV gene from the PRT strain of IHNV did not result in any significant change in viral growth. Deletion of the ^32^EGDL^35^ resulted in a 9- and 5-fold decrease in growth (9.8×10^4^ and 7.8×10^5^ PFU/ml) relative to rIHNV-NV_PRT_ (8.9×10^5^ and 3.9×10^6^ PFU/ml) at 24 and 48 h p.i., respectively. Deletion of the entire NV gene resulted in a 9- and 12-fold decrease in growth (9.8×10^4^ and 3.1×10^5^ PFU/ml) relative to the parental rIHNV-32/87 (8.6×10^5^ and 3.8×10^6^ PFU/ml) at 24 and 48 h p.i., respectively. To determine whether expression of wild-type NV rescues the growth of NV-knockout rIHNV, we analyzed the growth of rIHNVs in RTG-2 cells transfected with pcDNA3/NV_PRT_. As shown in Fig. 5C, overexpression of NV significantly increased the viral yields of rIHNV-32/87-ΔNV-GFP. These results demonstrate that NV is essential for efficient growth of IHNV in RTG-2 cells and, importantly, that the amino acid sequence ^32^EGDL^35^ is critical for the functioning of NV. However, deletion of the entire NV leads to a greater decrease in viral growth than deletion of only the ^32^EGDL^35^. After 72 h p.i., even though the viral yield of all four rIHNVs increased, the difference in viral yield among the four rIHNVs decreased and at 120 h p.i., all four rIHNVs yielded a similar final infectivity titer (6.7∼7.4 10^7^ PFU/ml) (data not shown). The reduced difference after 72 h p.i. was likely because the RTG-2 cells infected with rIHNV-32/87 or rIHNV-NV_PRT_ were largely dead by 72 h p.i. while most cells infected with rIHNV-32/87-ΔNV-GFP or rIHNV-NV_PRT_-ΔEGDL remained alive.

### NV is required for inhibition of poly I∶C-induced IFN responses in RTG-2 cells

**Figure 6:**
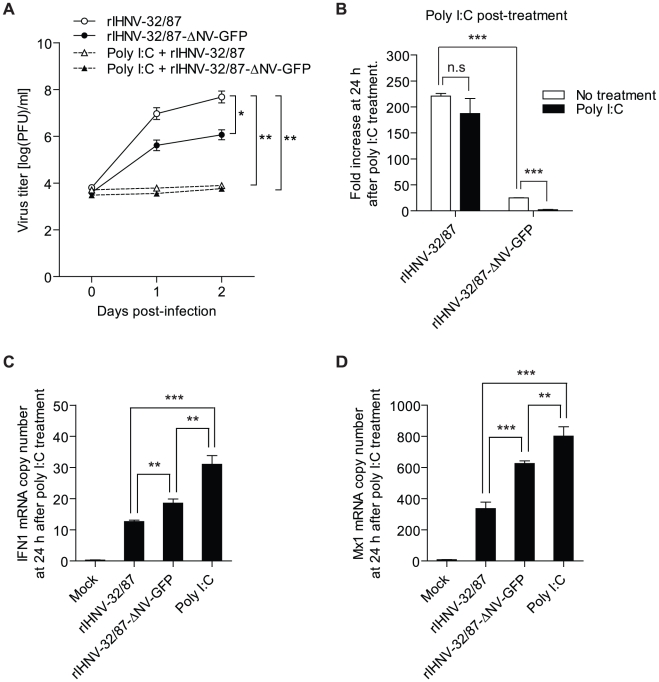
Requirement of NV for inhibition of poly I∶C-induced IFN response in RTG-2 cells. *A*, Effect of poly I∶C pre-treatment on the growth of rIHNVs. RTG-2 cells were pre-incubated with poly I∶C at 25 µg/ml for 24 h. The cells were then infected with rIHNV-32/87 or rIHNV-32/87-ΔNV-GFP at an MOI of 0.01 PFU/cell and samples of the supernatant medium were collected at 0, 24, and 48 h p.i. All data points represent the average of samples taken from duplicate infections. *B*, Effect of poly I∶C treatment after virus infection on the growth of rIHNVs. RTG-2 cells were infected with rIHNV-32/87 or rIHNV-32/87-ΔNV-GFP at an MOI of 0.01 PFU/cell. After incubation for 24 h, cells were washed three times and treated with 25 µg/ml of poly I∶C or serum-free media. At 0 and 24 h after poly I∶C treatment, samples of the supernatant medium were collected and titrated in duplicate. The virus titer in the supernatant medium collected at 0 h after poly I∶C treatment was defined as one. The results are presented as the means ± SD of three independent experiments (***P<0.001). ns, not significant. *C* and *D*, Analysis of IFN1 and Mx1 expressions in RTG-2 cells treated with poly I∶C after virus infection. RTG-2 cells were infected with rIHNV-32/87 or rIHNV-32/87-ΔNV-GFP at an MOI of 1 PFU/cell. After incubation for 24 h, cells were incubated with culture media containing 25 µg/ml of poly I∶C. At 24 h after poly I∶C treatment, total RNA was extracted from the cells and analyzed with real-time PCR for IFN1 (*C*) and Mx1 (*D*). The negative control was mock-infected RTG-2 cells without poly I∶C treatment. The positive control was mock-infected RTG-2 cells stimulated with 25 µg/ml poly I∶C. The levels of IFN1 and Mx1 are expressed as mRNA copy number normalized to 1000 copies of ARP mRNA. The results are presented as the means ± SD of three independent experiments (**P<0.01; ***P<0.001).

Poly I∶C is a well-documented inducer of IFN systems and viral resistance in animal cells [43]. To determine the effects of a poly I∶C-induced IFN response on the growth of the rIHNVs, RTG-2 cells were treated with 25 µg/ml of poly I∶C. As shown in the poly I∶C positive controls in Figure 6C and 6D, this treatment significantly induced the expression of both IFN1 and the Mx1 gene in RTG-2 cells at 24 h after treatment. At 24 h after poly I∶C treatment cells were infected with rIHNV-32/87 or rIHNV-32/87-ΔNV-GFP, samples of culture supernatant were collected at 0, 24, and 48 h p.i., and infectious titers were determined by plaque assay. Poly I∶C pre-treatment dramatically blocked the growth of both rIHNV-32/87 and rIHNV-32/87-ΔNV-GFP (Figure 6A). These results suggest that pre-existing antiviral responses induced by poly I∶C in RTG-2 cells can block the growth of IHNV even with a functional NV.

Next, we determined whether poly I∶C treatment after viral infection can affect the growth of the IHNV. RTG-2 cells were infected with rIHNV-32/87 or rIHNV-32/87-ΔNV-GFP and then treated at 24 h p.i. with 25 µg/ml of poly I∶C. The culture supernatants were collected at 0 and 24 h after poly I∶C treatment and infectious titers were determined by plaque assay. As shown in Figure 6B, while the growth of wild-type rIHNV-32/87 was not significantly affected (1.2-fold), poly I∶C treatment post-infection induced a 13-fold decrease in the yield of the NV-knockout mutant rIHNV-32/87-ΔNV-GFP. This suggests that wild type IHNV may have mechanisms for overcoming the antiviral activity induced by poly I∶C 24 h after IHNV infection, and that the NV may be the key factor for this function.

To test if NV is involved in inhibition of the IFN response induced by poly I∶C, RTG-2 cells were infected with rIHNV-32/87 or rIHNV-32/87-ΔNV-GFP and then treated at 24 h p.i. with 25 µg/ml of poly I∶C. Cells were collected at 24 h after poly I∶C treatment and the expression levels of IFN1 and Mx1 were determined using real-time PCR. Results were expressed as mRNA copy number and normalized per 1000 copies of a housekeeping gene mRNA (ARP). Cells infected with wild-type rIHNV-32/87 or NV-knockout mutant rIHNV-32/87-ΔNV-GFP produced decreased levels of IFN1 (12 and 19 copies, respectively) and Mx1 (327 and 625 copies, respectively) compared with mock-infected poly I∶C treated control (IFN1, 31 copies; Mx1, 801 copies). However, cells infected with rIHNV-32/87-ΔNV-GFP produced 1.4- and 1.8-fold higher amount of IFN1 and Mx1, respectively, than those infected with rIHNV-32/87 (Figure 6C, 6D). This suggests that NV is required for effective inhibition of poly I∶C-induced IFN responses in RTG-2 cells, but virus without NV is also capable of significant inhibition.

### NV is required for the inhibition of the IHNV-induced IFN response in RTG-2 cells

**Figure 7:**
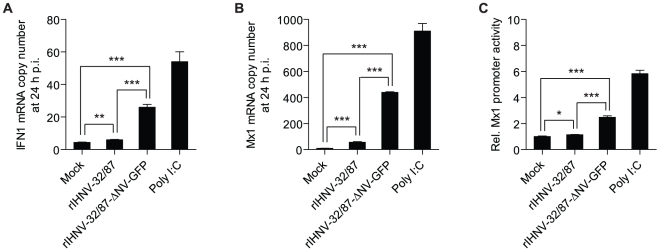
Requirement of NV for inhibition of IFN systems in IHNV-infected RTG-2 cells. *A* and *B*, IFN1 and Mx1 expression in RTG-2 cells infected with rIHNV-32/87 or rIHNV-32/87-ΔNV-GFP. RTG-2 cells were harvested at 24 h p.i., and total RNA was analyzed with real-time PCR for IFN1 (*A*) and Mx1 (*B*). The negative control was mock-infected RTG-2 cells without poly I∶C treatment. The positive control was mock-infected RTG-2 cells stimulated with 25 µg/ml poly I∶C. The levels of IFN1 and Mx1 are expressed as mRNA copy number normalized to 1000 copies of ARP mRNA. The results are presented as the means ± SD of three independent experiments (**P<0.01; ***P<0.001). *C*, Assay for functional IFN1 released from RTG-2 cells infected with rIHNV-32/87 or rIHNV-32/87-ΔNV-GFP. The culture supernatants were collected at 24 h p.i. and IFN activities were measured by adding the supernatants to RTG-P1 cells and analyzing luciferase activity in RTG-P1 cells as described in the Materials and Methods. The negative control was mock-infected RTG-2 cells without poly I∶C treatment. The positive control was mock-infected RTG-2 cells stimulated with 25 µg/ml poly I∶C. The luciferase activity in the RTG-P1 cells treated with supernatants from the negative control cells was defined as one. The results are presented as the means ± SD of three independent experiments (*P<0.05; ***P<0.001).

We also determined whether NV is involved in the blocking of the IHNV-induced IFN system. RTG-2 cells were infected with rIHNV-32/87 or rIHNV-32/87-ΔNV-GFP, after which total RNA was extracted at 24 h p.i., and the induction of IFN1 was analyzed using real-time RT-PCR. While wild-type rIHNV-32/87 induced a 1.5-fold increase in IFN1 expression (6 copies per 1000 copies of ARP mRNA) compared with mock-infected controls (4 copies), the NV-knockout mutant rIHNV-32/87-ΔNV-GFP induced a 6-fold increase in IFN1 expression (25 copies) (Figure 7A). If the NV is involved in the inhibition of IFN1 expression in IHNV-infected cells, the NV-knockout mutant rIHNV should induce higher levels of interferon-stimulated genes (ISGs) than the wild-type rIHNV. To test this, we analyzed the expression level of the Mx1 gene, one of the ISGs, in the rIHNV-infected RTG-2 cells using real-time PCR. While wild-type rIHNV-32/87 induced a 6-fold increase in Mx1 gene expression (64 copies) relative to mock infected controls (10 copies), the NV-knockout mutant rIHNV-32/87-ΔNV-GFP induced a 48-fold increase of in Mx1 gene expression (478 copies) (Figure 7B). Taken together, these data indicate that the NV may support the growth of IHNV through inhibition of the IFN system in IHNV-infected RTG-2 cells.

In addition, we tested the effect of NV on the secretion of functional IFN1 from IHNV-infected RTG-2 cells. Supernatants were collected from RTG-2 cells infected with rIHNV-32/87 or rIHNV-32/87-ΔNV-GFP at 24 h p.i. and IFN1 activities in the supernatants were assayed using RTG-P1 cells. RTG-P1 cells are RTG-2 derivatives stably transfected with a luciferase reporter vector controlled by a rainbow trout Mx1 promoter [36]. RTG-P1 cells were treated with supernatants of RTG-2 cells for 48 h, cell extracts were prepared and luciferase activity was measured. RTG-P1 cells treated with supernatant from mock-infected RTG-2 cells were used as a negative control and those treated with 25 µg/ml of poly I∶C were used as a positive control. While the supernatant of wild-type rIHNV-32/87-infected RTG-2 cells showed a 1.1-fold increases in luciferase activity compared with that of mock-infected RTG-2 cells, that of NV-knockout mutant rIHNV-32/87-ΔNV-GFP induced a 2.5-fold increase in luciferase activity (Figure 7C). This result is similar to the IFN1 gene expression pattern obtained using real-time PCR (Figure 7A). Together, these data revealed that the NV plays an important role in the inhibition of IFN1 expression in IHNV-infected RTG-2 cells.

### Amino acid residues ^32^EGDL^35^ are essential for the anti-IFN activity of NV

**Figure 8:**
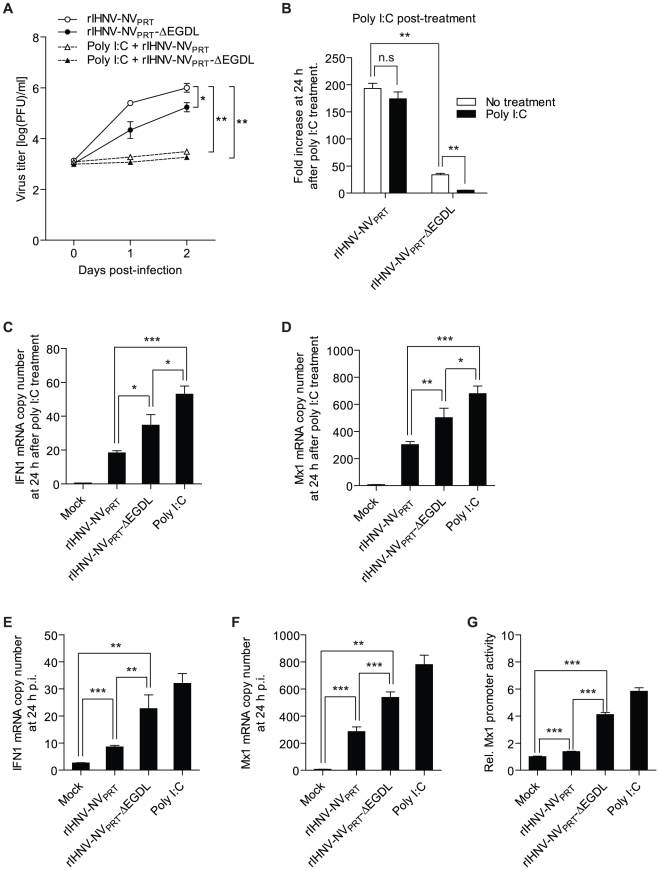
The amino acids ^32^EGDL^35^ are essential for the IFN1 inhibitory function of NV. *A*, Effects of poly I∶C pre-treatment on the growth of rIHNV-NV_PRT_ and rIHNV-NV_PRT_-ΔEGDL in RTG-2 cells. RTG-2 cells were pre-incubated with poly I∶C at 25 µg/ml for 24 h. Cells were then infected with rIHNV-NV_PRT_ or rIHNV-NV_PRT_-ΔEGDL at an MOI of 0.01 PFU/cell, and samples of the supernatant medium were collected at 0, 24, and 48 h p.i. All data points represent the average titers of samples taken from duplicate infections. *B*, Effects of poly I∶C treatment after viral infection on the growth of rIHNV-NV_PRT_ and rIHNV-NV_PRT_-ΔEGDL. RTG-2 cells were infected with rIHNV-NV_PRT_ and rIHNV-NV_PRT_-ΔEGDL at an MOI of 0.01 PFU/cell. After incubation for 24 h, cells were washed and incubated with fresh culture media containing 25 µg/ml of poly I∶C. At 0 and 24 h after poly I∶C treatment, samples of the supernatant medium were collected. The virus titer in the supernatant medium collected at 0 h after poly I∶C treatment was defined as one. The results are presented as the means ± SD of three independent experiments (**P<0.01). ns, not significant. *C* and *D*, Analysis of IFN1 and Mx1 expressions in RTG-2 cells treated with poly I∶C after viral infection. RTG-2 cells were infected with rIHNV-NV_PRT_, or rIHNV-NV_PRT_-ΔEGDL at an MOI of 1 PFU/cell. After incubation for 24 h, cells were incubated with culture media containing 25 µg/ml of poly I∶C. At 24 h after poly I∶C treatment, total RNA was extracted from the cells and analyzed with real-time PCR for IFN1 (*C*) and Mx1 (*D*). The negative control was mock-infected RTG-2 cells without poly I∶C treatment. The positive control was mock-infected RTG-2 cells stimulated with 25 µg/ml poly I∶C. The levels of IFN1 and Mx1 are expressed as mRNA copy number normalized to 1000 copies of ARP mRNA. The results are presented as the means ± SD of three independent experiments (*P<0.05; **P<0.01; ***P<0.001). *E* and *F*, IFN1 and Mx1 expression in RTG-2 cells infected with rIHNV-NV_PRT_, or rIHNV-NV_PRT_-ΔEGDL. RTG-2 cells were harvested at 24 h p.i., and the total RNA was analyzed with real-time PCR for IFN1 (*E*) and Mx1 (*F*). The negative control was mock-infected RTG-2 cells without poly I∶C treatment. The positive control was mock-infected RTG-2 cells stimulated with 25 µg/ml poly I∶C. The levels of IFN1 and Mx1 are expressed as mRNA copy number normalized to 1000 copies of ARP mRNA. The results are presented as the means ± SD of three independent experiments (**P<0.01; ***P<0.001). *G*, Assay for functional IFN1 released from RTG-2 cells infected with rIHNV-NV_PRT_, or rIHNV-NV_PRT_-ΔEGDL. The culture supernatants were collected from RTG-2 cells infected with rIHNV-NV_PRT_, or rIHNV-NV_PRT_-ΔEGDL at 24 h p.i. The IFN1 activities in the supernatants were measured as described in the legend of Figure 7C. The negative control was mock-infected RTG-2 cells without poly I∶C treatment. The positive control was mock-infected RTG-2 cells stimulated with 25 µg/ml poly I∶C. The luciferase activity in the RTG-P1 cells treated with supernatants from the negative control cells was defined as one. The results are presented as the means ± SD of three independent experiments (***P<0.001).

Amino acids ^32^EGDL^35^ within the NV were found to be required for the efficient growth of IHNV in RTG-2 cells (Figure 5B). Therefore, it is possible that this amino acid sequence is essential for the ant-IFN activity of NV. We first determined whether ^32^EGDL^35^ are required for efficient growth of IHNV in poly I∶C-treated RTG-2 cells. In RTG-2 cells pre-treated with 25 µg/ml of poly I∶C for 24 h, the growth of both rIHNV-NV_PRT_ and rIHNV-NV_PRT_-ΔEGDL were blocked (Figure 8A). However, in RTG-2 cells treated with poly I∶C at 24 h after viral infection, the growth of wild-type rIHNV-NV_PRT_ was not significantly affected by poly I∶C treatment, while that of rIHNV-NV_PRT_-ΔEGDL was severely decreased (Figure 8B). Thus amino acids ^32^EGDL^35^ are required for efficient growth of IHNV in RTG-2 cells treated with poly I∶C after virus infection. We also determined the expression levels of IFN1 and Mx1 in RTG-2 cells which were infected with rIHNV-NV_PRT_ or rIHNV-NV_PRT_-ΔEGDL and then treated with 25 µg/ml of poly I∶C at 24 h p.i. Cells infected with rIHNV-NV_PRT_ or rIHNV-NV_PRT_-ΔEGDL produced decreased levels of IFN1 (18 and 35 copies, respectively) and Mx1 (290 and 503 copies, respectively) compared with mock-infected poly I∶C treated control (IFN1, 53 copies; Mx1, 680 copies). However, cells infected with rIHNV-NV_PRT_-ΔEGDL produced 1.9- and 1.7-fold higher amount of IFN1 and Mx1, respectively, than those infected with rIHNV-NV_PRT_ (Figure 8C, 8D).

To determine whether ^32^EGDL^35^ are required to inhibit the host IFN system, RTG-2 cells were infected with rIHNV-NV_PRT_ or rIHNV-NV_PRT_-ΔEGDL and the expression levels of IFN1 and Mx1 were analyzed using real-time PCR at 24 h p.i. Their mRNA copy numbers were normalized per 1000 copies of ARP mRNA. While rIHNV-NV_PRT_ induced 3- and 43-fold increases in IFN1 (9 copies) and Mx1 (295 copies) expressions compared with mock-infected control (IFN1, 3 copies; Mx1, 7 copies), respectively, rIHNV-NV_PRT_-ΔEGDL induced 9-fold and 73-fold increases of IFN1 (26 copies) and Mx1 (515 copies) expressions, respectively (Figure 8E, 8F). In addition, RTG-P1 cells treated with supernatants from rIHNV-NV_PRT_-ΔEGDL-infected RTG-2 cells showed 3.0-fold higher level of luciferase activity than those treated with supernatants from rIHNV-NV_PRT_-infected RTG-2 cells (Figure 8G). Collectively, these data demonstrate that the amino acid sequence ^32^EGDL^35^ is essential for the efficient anti-IFN activity of NV.

## Discussion

NV is a non-virion protein whose gene is unique to the genus *Novirhabdovirus*. Although NV is known to be required for the efficient growth of IHNV in rainbow trout [6], the exact role of the NV in IHNV infection is still unclear. In this study, we investigated the cellular localization of NV by transient expression of a NV-GFP fusion protein and showed that the NV protein can be actively imported into the nucleus, since it can direct nuclear accumulation of a cytoplasmic protein, GFP, in transiently transfected CHSE-214 and RTG-2 cells. This demonstrates that, even though it is smaller than the diffusion limits of the nuclear pore complex (NPC), the 111-aa NV protein of IHNV is able to exploit cellular mechanisms for active nuclear import. The region of NV protein responsible for nuclear localization was mapped to amino acid residues ^32^EGDL^35^. A survey of IHNV NV sequences present in GenBank revealed that these residues were conserved among IHNV strains and especially amino acid residues ^33^GDL^35^ were strictly conserved among all IHNV strains of which NV has been sequenced (data not shown). We eliminated the possibility that this short amino acid region comprises a portion of a larger signal disrupted by removal of these amino acids, since the addition of amino acids ^32^EGDL^35^ to GFP resulted in the accumulation of fluorescent label in the nucleus. To determine the role of this motif in IHNV replication, we constructed a mutant rIHNV expressing a ^32^EGDL^35^-deleted NV protein (rIHNV-NV_PRT_-ΔEGDL) and compared its growth in rainbow trout cells with that of an NV-knockout mutant rIHNV (rIHNV-32/87-ΔNV-GFP). Even though there was a slight difference in their growth, the growth of both mutant rIHNVs was highly attenuated in RTG-2 cells relative to rIHNV with an intact NV gene, indicating that ^32^EGDL^35^-mediated nuclear localization of NV is important for NV function.

Importin β (karyopherin) proteins bind nuclear localization signals (NLSs) to facilitate the nuclear import of NLS-containing proteins [41]. Interestingly, amino acid sequence analysis revealed that the IHNV NV does not contain any classical NLS. Therefore it is necessary to consider the mechanism by which the NV protein enters the nucleus. One possibility is that the amino acid sequence ^32^EGDL^35^ is a novel NLS which can mediate the nuclear transport of NV via binding to importins. Generally, NLSs which bind either importin α or importin β have basic amino acid residues such as lysine at their cores [41], [44]. However, ^32^EGDL^35^ do not contain any basic residues but, on the contrary, possess acidic residues such as glutamic acid (E) and aspartic acid (D). Thus, it is unlikely that ^32^EGDL^35^ bind directly to importins. Another possibility is that this motif mediates the nuclear import of NV by binding to other proteins containing NLSs. Even though the amino acid sequence ^32^EGDL^35^ is highly conserved among IHNV strains, it does not show any similarity to other known motifs or domains. Thus, at present, it is difficult to predict candidates for NLS-containing proteins to which ^32^EGDL^35^ can bind. Further characterization of the NV-interacting proteins, including importins, will be needed to elucidate the molecular mechanism through which the NV protein enters the nucleus.

Type I IFNs are well-known to block virus multiplication by inducing upregulation of antiviral genes [8], [9]. Recently, IFN genes have been identified in a number of species of teleost fish including rainbow trout [10]. Thus, IHNV should possess strategies to block the induction of IFN systems in trout RTG-2 cells which would otherwise suppress virus growth early in infection [7]. The nonstructural proteins of RNA viruses have been reported to be implicated in down-regulation of the host innate immune response [7], [45]. In this study, we provided evidences that the IHNV NV possesses capability for down-regulation of the host IFN system: (a) NV-knockout mutant rIHNV induced greater expressions of the IFN1 and Mx1 genes than the wild-type rIHNV in RTG-2 cells; (b) RTG-2 cells infected with NV-knockout rIHNV produced a higher level of IFN1 activity in cell culture supernatant relative to wild-type rIHNV; (c) wild-type rIHNV more efficiently blocked the expressions of IFN1 and Mx1 than the NV-knockout mutant rIHNV in RTG-2 cells treated with poly I∶C after viral infection. These results suggest that the NV protein can support IHNV growth through inhibition of host IFN systems.

It is next necessary to determine the role of nuclear localization of the NV as it pertains to its inhibitory function. IHNV is an RNA virus that is believed to replicate in the cytoplasm like other RNA viruses. Many RNA viruses whose primary site of replication is the cytoplasm produce viral proteins that localize to the nucleus during virus infection in order to inhibit IFN systems [46]. Thus, it is possible to speculate that the IHNV NV protein localizes to the nucleus to block the induction of IFN systems in RTG-2 cells. If this is true, we would expect that the mutant rIHNV expressing an ^32^EGDL^35^-deleted NV protein would less effectively block the IFN systems. Consistent with our expectation, an rIHNV mutant with an ^32^EGDL^35^-deleted NV induced more up-regulations of IFN1 and Mx1 gene expressions than the wild-type rIHNV in RTG-2 cells, and cells infected with this mutant produced a higher level of IFN1 activity in cell culture supernatant relative to wild-type rIHNV-infected cells. In addition, this mutant failed to block the inductions of IFN1 and Mx1 in RTG-2 cells treated with poly I∶C after viral infection. The ^32^EGDL^35^ deletion mutant mimicked the NV knockout rIHNV in all experiments conducted here, suggesting that the ^32^EGDL^35^-mediated nuclear localization of NV is critical for the inhibitory function of NV.

The existence of the NV gene is restricted only to the genus *Novirhabdovirus* and, thus, it is likely that rhabdoviruses belonging to genera other than the genus *Novirhabdovirus* use strategies different from that of IHNV; the rhabdovirus vesicular stomatitis virus (VSV) and rabies virus (RV) are good examples [24]. In the case of VSV, the matrix (M) protein contains NLSs [31] and is reported to suppress IFN systems through inhibition of the transcription factor TFIID [25], [26] and blocking of nuclear transport [29], [30], [35]. On the contrary, RV uses phosphoprotein (P) to inhibit host IFN systems [24]. The RV P protein contains an NLS [47] and suppresses the IFN systems through inhibition of the nuclear accumulation of STAT1, the binding of STAT1 to the DNA, and the function of ISG products such as PML [34], [48]. In this study, we did not determine the subcellular localization of IHNV M and P proteins and their potential functional roles in the inhibition of host IFN systems. However, it is possible to speculate that IHNV has evolved to use the NV protein instead of the M or P proteins to inhibit the host IFN systems.

Among other species in the genus *Novirhabdovirus* there are inconsistent reports regarding the importance of NV. For Viral hemorrhagic septicemia virus (VHSV) recent reports demonstrate that NV is required for efficient viral growth in cell culture and pathogenicity in fish [49], [50]. VHSV NV was reported to rescue the growth of NV-knockout mutant rIHNV [6], suggesting the possibility that VHSV NV supports the viral growth via inhibition of host IFN system as IHNV NV does. However, the underlying mechanisms of IFN inhibition of VHSV NV may be different from those of IHNV NV, since VHSV NV exhibits a low level of amino acid sequence similarity with IHNV NV and does not have EGDL motif. It is unclear whether all novirhabdoviruses use the NV to inhibit IFN systems, but if they do, a different NLS may be utilized. In the case of a different species, snakehead rhabdovirus (SHRV), an NV-knockout mutant virus showed the same pathogenicity as wild-type virus, suggesting that this virus may cope with the host antiviral response using proteins other than NV [51]. Pathogenicity of SHRV was tested using zebrafish instead of snakehead. Thus, it is possible that the NV of SHRV might play an essential role for the efficient growth and pathogenicity of the virus in the snakehead fish host from which it originated.

Our data revealed that, in RTG-2 cells, poly I∶C treatment before viral infection induced high levels of IFN1 and Mx1 and limited production of both wild-type and NV-knockout rIHNV. However, when poly I∶C treatment occurred after infection, this treatment did not affect the growth of wild-type rIHNV but significantly limited that of the NV-knockout rIHNV. This suggests that, in RTG-2 cells treated with poly I∶C after IHNV infection, wild-type IHNV but not NV-knockout mutant IHNV can cope with the IFN systems using the NV. However, in RTG-2 cells pre-treated with poly I∶C, pre-existing antiviral activities may block the expression of viral genes and, thus, it may be impossible for IHNV to express a sufficiently large amount of NV protein adequate to inhibit IFN systems.

In conclusion, our results show that the NV protein localizes to the nucleus, and that the amino acid sequence ^32^EGDL^35^ within the NV protein is responsible for nuclear localization. Even though the NV protein cannot cope with a pre-existing strong IFN response, it has the capability to inhibit IFN1 synthesis and ^32^EGDL^35^ are essential for this inhibitory activity. It remains unclear as to how ^32^EGDL^35^ mediate the nuclear localization of NV and how these residues inhibit IFN systems. It will be interesting to know which cellular proteins recognize these residues of the NV protein to mediate nuclear localization of NV and to inhibit IFN systems.

## References

[1] Bootland LM, Leong JC, Woo PTK, Bruno DW (1999). Infectious hematopoietic necrosis virus;.

[2] Wolf K (1988). Infectious hematopoietic necrosis.

[3] Kurath G, Ahern KG, Pearson GD, Leong JC (1985). Molecular cloning of the six mRNA species of infectious hematopoietic necrosis virus, a fish rhabdovirus, and gene order determination by R-loop mapping.. J Virol.

[4] Morzunov SP, Winton JR, Nichol ST (1995). The complete genome structure and phylogenetic relationship of infectious hematopoietic necrosis virus.. Virus Res.

[5] Biacchesi S, Thoulouze MI, Bearzotti M, Yu YX, Bremont M (2000). Recovery of NV knockout infectious hematopoietic necrosis virus expressing foreign genes.. J Virol.

[6] Thoulouze MI, Bouguyon E, Carpentier C, Bremont M (2004). Essential role of the NV protein of Novirhabdovirus for pathogenicity in rainbow trout.. J Virol.

[7] Haller O, Kochs G, Weber F (2006). The interferon response circuit: induction and suppression by pathogenic viruses.. Virology.

[8] Sadler AJ, Williams BR (2008). Interferon-inducible antiviral effectors.. Nat Rev Immunol.

[9] Biron CA, Sen GC, DM K, PM H (2001). Interferons and other cytokines;.

[10] Robertsen B (2006). The interferon system of teleost fish.. Fish Shellfish Immunol.

[11] Robertsen B (2008). Expression of interferon and interferon-induced genes in salmonids in response to virus infection, interferon-inducing compounds and vaccination.. Fish Shellfish Immunol.

[12] Collet B, Secombes CJ (2002). Type I-interferon signalling in fish.. Fish Shellfish Immunol.

[13] Trobridge GD, Chiou PP, Leong JA (1997). Cloning of the rainbow trout (Oncorhynchus mykiss) Mx2 and Mx3 cDNAs and characterization of trout Mx protein expression in salmon cells.. J Virol.

[14] Saint-Jean SR, Perez-Prieto SI (2006). Interferon mediated antiviral activity against salmonid fish viruses in BF-2 and other cell lines.. Vet Immunol Immunopathol.

[15] Ooi EL, Verjan N, Haraguchi I, Oshima T, Kondo H (2008). Innate immunomodulation with recombinant interferon-alpha enhances resistance of rainbow trout (Oncorhynchus mykiss) to infectious hematopoietic necrosis virus.. Dev Comp Immunol.

[16] Guo Z, Chen LM, Zeng H, Gomez JA, Plowden J (2007). NS1 protein of influenza A virus inhibits the function of intracytoplasmic pathogen sensor, RIG-I.. Am J Respir Cell Mol Biol.

[17] Mibayashi M, Martinez-Sobrido L, Loo YM, Cardenas WB, Gale M (2007). Inhibition of retinoic acid-inducible gene I-mediated induction of beta interferon by the NS1 protein of influenza A virus.. J Virol.

[18] Opitz B, Rejaibi A, Dauber B, Eckhard J, Vinzing M (2007). IFNbeta induction by influenza A virus is mediated by RIG-I which is regulated by the viral NS1 protein.. Cell Microbiol.

[19] Ludwig S, Wang X, Ehrhardt C, Zheng H, Donelan N (2002). The influenza A virus NS1 protein inhibits activation of Jun N-terminal kinase and AP-1 transcription factors.. J Virol.

[20] Talon J, Salvatore M, O'Neill RE, Nakaya Y, Zheng H (2000). Influenza A and B viruses expressing altered NS1 proteins: A vaccine approach.. Proc Natl Acad Sci U S A.

[21] Wang X, Li M, Zheng H, Muster T, Palese P (2000). Influenza A virus NS1 protein prevents activation of NF-kappaB and induction of alpha/beta interferon.. J Virol.

[22] Greenspan D, Palese P, Krystal M (1988). Two nuclear location signals in the influenza virus NS1 nonstructural protein.. J Virol.

[23] Li Y, Yamakita Y, Krug RM (1998). Regulation of a nuclear export signal by an adjacent inhibitory sequence: the effector domain of the influenza virus NS1 protein.. Proc Natl Acad Sci U S A.

[24] Rieder M, Conzelmann KK (2009). Rhabdovirus evasion of the interferon system.. J Interferon Cytokine Res.

[25] Ahmed M, McKenzie MO, Puckett S, Hojnacki M, Poliquin L (2003). Ability of the matrix protein of vesicular stomatitis virus to suppress beta interferon gene expression is genetically correlated with the inhibition of host RNA and protein synthesis.. J Virol.

[26] Black BL, Lyles DS (1992). Vesicular stomatitis virus matrix protein inhibits host cell-directed transcription of target genes in vivo.. J Virol.

[27] Brzozka K, Finke S, Conzelmann KK (2005). Identification of the rabies virus alpha/beta interferon antagonist: phosphoprotein P interferes with phosphorylation of interferon regulatory factor 3.. J Virol.

[28] Brzozka K, Finke S, Conzelmann KK (2006). Inhibition of interferon signaling by rabies virus phosphoprotein P: activation-dependent binding of STAT1 and STAT2.. J Virol.

[29] Enninga J, Levy DE, Blobel G, Fontoura BM (2002). Role of nucleoporin induction in releasing an mRNA nuclear export block.. Science.

[30] Faria PA, Chakraborty P, Levay A, Barber GN, Ezelle HJ (2005). VSV disrupts the Rae1/mrnp41 mRNA nuclear export pathway.. Mol Cell.

[31] Glodowski DR, Petersen JM, Dahlberg JE (2002). Complex nuclear localization signals in the matrix protein of vesicular stomatitis virus.. J Biol Chem.

[32] Gustin KE, Sarnow P (2002). Inhibition of nuclear import and alteration of nuclear pore complex composition by rhinovirus.. J Virol.

[33] Vidy A, Chelbi-Alix M, Blondel D (2005). Rabies virus P protein interacts with STAT1 and inhibits interferon signal transduction pathways.. J Virol.

[34] Vidy A, El Bougrini J, Chelbi-Alix MK, Blondel D (2007). The nucleocytoplasmic rabies virus P protein counteracts interferon signaling by inhibiting both nuclear accumulation and DNA binding of STAT1.. J Virol.

[35] von Kobbe C, van Deursen JM, Rodrigues JP, Sitterlin D, Bachi A (2000). Vesicular stomatitis virus matrix protein inhibits host cell gene expression by targeting the nucleoporin Nup98.. Mol Cell.

[36] Collet B, Boudinot P, Benmansour A, Secombes CJ (2004). An Mx1 promoter-reporter system to study interferon pathways in rainbow trout.. Dev Comp Immunol.

[37] Park MA, Sohn SG, Lee SD, Chun SK, Park JW (1993). Infectious haematopoietic necrosis virus from salmonids cultured in Korea.. Journal of Fish Diseases.

[38] Batts WN, Winton JR (1989). Enhanced detection of infectious hematopoietic necrosis virus and other fish viruses by pretreatment of cell monolayers with polyethylene glycol.. Journal of Aquatic Animal Health.

[39] Fuerst TR, Niles EG, Studier FW, Moss B (1986). Eukaryotic transient-expression system based on recombinant vaccinia virus that synthesizes bacteriophage T7 RNA polymerase.. Proc Natl Acad Sci U S A.

[40] Purcell MK, Kurath G, Garver KA, Herwig RP, Winton JR (2004). Quantitative expression profiling of immune response genes in rainbow trout following infectious haematopoietic necrosis virus (IHNV) infection or DNA vaccination.. Fish Shellfish Immunol.

[41] Lange A, Mills RE, Lange CJ, Stewart M, Devine SE (2007). Classical nuclear localization signals: definition, function, and interaction with importin alpha.. J Biol Chem.

[42] Chook YM, Blobel G (2001). Karyopherins and nuclear import.. Curr Opin Struct Biol.

[43] Field AK, Tytell AA, Lampson GP, Hilleman MR (1967). Inducers of interferon and host resistance. II. Multistranded synthetic polynucleotide complexes.. Proc Natl Acad Sci U S A.

[44] Lee BJ, Cansizoglu AE, Suel KE, Louis TH, Zhang Z (2006). Rules for nuclear localization sequence recognition by karyopherin beta 2.. Cell.

[45] Garcia-Sastre A, Biron CA (2006). Type 1 interferons and the virus-host relationship: a lesson in detente.. Science.

[46] Hiscox JA (2003). The interaction of animal cytoplasmic RNA viruses with the nucleus to facilitate replication.. Virus Res.

[47] Pasdeloup D, Poisson N, Raux H, Gaudin Y, Ruigrok RW (2005). Nucleocytoplasmic shuttling of the rabies virus P protein requires a nuclear localization signal and a CRM1-dependent nuclear export signal.. Virology.

[48] Chelbi-Alix MK, Vidy A, El Bougrini J, Blondel D (2006). Rabies viral mechanisms to escape the IFN system: the viral protein P interferes with IRF-3, Stat1, and PML nuclear bodies.. J Interferon Cytokine Res.

[49] Ammayappan A, Kurath G, Thompson TM, Vakharia VN (2010). A Reverse Genetics System for the Great Lakes Strain of Viral Hemorrhagic Septicemia Virus: the NV Gene is Required for Pathogenicity.. Mar Biotechnol (NY).

[50] Biacchesi S, Lamoureux A, Merour E, Bernard J, Bremont M (2010). Limited interference at the early stage of infection between two recombinant novirhabdoviruses: viral hemorrhagic septicemia virus and infectious hematopoietic necrosis virus.. J Virol.

[51] Alonso M, Kim CH, Johnson MC, Pressley M, Leong JA (2004). The NV gene of snakehead rhabdovirus (SHRV) is not required for pathogenesis, and a heterologous glycoprotein can be incorporated into the SHRV envelope.. J Virol.

